# Cognitive Enhancement, Hyperagency, and Responsibility Explosion

**DOI:** 10.1093/jmp/jhae025

**Published:** 2024-06-24

**Authors:** Emma C Gordon

**Affiliations:** University of Glasgow, Glasgow, United Kingdom

**Keywords:** *cognitive enhancement*, *hyperagency*, *responsibility*

## Abstract

*Hyperagency objections appeal to the risk that cognitive enhancement may negatively impact our well-being by giving us too much control. I charitably formulate and engage with a prominent version of this objection due to* *Sandel (2009)* *—viz., that cognitive enhancement may negatively impact our well-being by creating an “explosion” of responsibilities. I first outline why this worry might look* prima facie *persuasive, and then I show that it can ultimately be defended against. At the end of the day, if we are to resist cognitive enhancement, it should not be based on a Sandel-style hyperagency argument.*

## I. INTRODUCTION

The human enhancement debate explores the ethics of using new and emerging drugs and other biotechnologies to improve aspects of ourselves and our lives^1^, from our cognitive capacities to our moral disposition^2^ and relationships^3^. For present purposes, we are interested in one particular subset of enhancement—*cognitive enhancement,* which aims to augment our cognitive capacities past the point of correcting pathology (Juengst and Moseley, 2016). More specifically, we consider one way in which the improvements associated with more radical forms of cognitive enhancement are—perhaps paradoxically—claimed to have a negative impact on our well-being^4^.

Imagine for a moment that you could effortlessly “level yourself up” in real life, in much the same way as you might level up a character in a video game with a few clicks of a button. Perhaps you have a harmless brain-computer interface (BCI) or neural implant^5^ implanted through a simple, noninvasive procedure, or perhaps you swallow a perfectly safe pill (i.e., one with no dangerous side effects) that immediately doubles your intelligence, your reasoning abilities, your information processing abilities, and your focus. In short, imagine that through the use of cognitive enhancement biotechnology, you are now cognitively “superhuman.” It is easy to see how, with this superhuman boost, your life could go better in many ways. For one thing, with all of these boosted capacities, you can more easily achieve your goals^6^. If you are a lawyer, you can now more easily win cases. If you are a chess player, you can see dozens or even hundreds of moves ahead. And if you are an oncologist, you can now diagnose cancer more quickly and more effectively design treatment protocols.

However, much more interesting than how your life could go better in such a circumstance is how your life could go *worse.* Along with acquiring all of this new power—including the power to continue upgrading your powers, altering what options are available—you at the same time acquire a lot more *responsibility* than you had before^7^. Whereas before, you were less responsible for how things go for yourself, your loved ones, and your wider community—given the limitations on what you were *able* to anticipate and do about these things in light of the capacities you were originally gifted^8^—these limitations (or at least some of them) have now been lifted. The worry here—which we call the *argument from hyperagency*—is primarily discussed in work by Michael Sandel (2009). Sandel’s thinking is that, perhaps counterintuitively, life for a superpowered person would inevitably be an unpleasant one, one that is marred by an “explosion” of responsibilities which in turn give rise to an explosion of unfulfilled desires. As Danaher (2014) puts it, when we think about what it means to have control over this many constitutive aspects of our agency, we see it is not welcome. And to the extent that cognitive enhancement gives us this control, it in this respect is a burden disguised as a blessing.

The following is a charitable reconstruction of Sandel’s hyperagency objection to enhancement, which takes the connection between hyperagency and an allegedly problematic “explosion of responsibility” as the driving idea, where this explosion of responsibility is taken to generate a “moral burden,” presumably (we may assume) one we are not equipped to meet, and on the relatively weak presumption that we would desire to do so (2007, 103–4). The reasoning (which makes implicit premises explicit, to make the reasoning valid) is as follows:

*Argument from Hyperagency*

(P1) The enhancement of capacities makes probable a corresponding increase of responsibilities for the results of one’s choices proportionate to the degree of the enhancement.

(P2) To the extent that the enhancement of capacities makes probable additional responsibilities for the results of one’s choices, it generates additional desires to fulfil these additional responsibilities.

(P3) If these additional desires are not fulfilled, well-being will be impeded.

(P4) It is more probable that, through the enhancement of capacities, an increase of responsibilities and desires to fulfil them is not accompanied by fulfilled desires than that it is.

(C1) Therefore, it is more probable that enhanced capacities will impede well-being than not.

(P5) If it is more probable that doing something,ϕ, will impede one’s well-being than not, then *ceteris paribus* one should notϕ.

(C2) Therefore, *ceteris paribus* one should not enhance one’s capacities.

Sandel’s hyperagency argument, part of his wider project in *The Case Against Perfection*, effectively subverts what we might initially be inclined to think about the relationship between our capabilities and our well-being. In this paper, I suggest that we ought to reject this kind of template version of the hyperagency argument (as well as another version of the argument I consider, which does not rely on (P2) and (P3)), and that, importantly, there are potentially several different routes to doing so, all of which press back against different aspects of the above reasoning. In Section II, I briefly note why (P3) and (P5) should both be granted—at least if we interpret them appropriately charitably. Thereafter, I turn to the three potentially contested premises—(P1), (P2), and (P4). In Section III, I evaluate the prima facie plausible (P1) and consider some further support for it, while also registering some potential concerns. Section IV then offers cause to doubt (P2) but ultimately suggests that all Sandel needs to do to accommodate my concerns is to *amend* his second premise in a way that does not involve any substantial concession. In Section V, I show why—perhaps contrary to initial appearances—the weak spot in the argument is actually (P4)—namely, the claim that it is more probable that, through the enhancement of capacities, an increase of responsibilities and desires to fulfill them is not accompanied by fulfilled desires than that it is. I show that this claim faces intractable objections based on which we should reject it, and further, that the underlying reasoning behind (P4) also generalizes in a problematic way. On the basis of these problems, the argument is unsound.

Section VI concludes by considering the prospects of running a version of the hyperagency argument that does not rely on P2 (or by extension P3), and that instead opts for a more direct connection between responsibility failure and well-being depletion. We see that on such a revision of the argument, there remains a problem with a (variation of) P4, and that the revised version of the argument accordingly faces structurally similar problems.

## II. WHY WE SHOULD ACCEPT P3 AND P5

Premise (3) of the argument is arguably the most plausible; an increase in unfulfilled desires—including desires to fulfill additional responsibilities, or to realize what we take to be exceptional potential—will very likely contribute to a depletion of well-being. We can see this result if we apply any one of a range of theories of well-being^9^. For example, if we look at hedonistic theories of well-being^10^—which tell us, roughly, that the more pleasure we experience the better off we are—we see that unfulfilled desires are very likely to induce *dis*pleasure, and thereby reduce well-being. And if we apply desire-fulfillment theories of well-being—which Heathwood notes are “nowadays undoubtedly one of the leading theories of well-being”—we have an even more straightforward explanation of how hyperagency depletes well-being (2017, 135).^11^ On such views, well-being is fundamentally a function of desire-fulfillment—and the unfulfilled desires of the hyperagent (e.g., to fulfill responsibilities, to realize seemingly unlimited potential, etc.) thereby immediately make a negative contribution to well-being.

For present purposes, I also think we can grant Sandel (P5)—i.e., the implicit premise that if it is more probable that doing something, ϕ, will impede one’s well-being than not, then *ceteris paribus* one should not do ϕ. It is important though to clarify what this premise is not saying. By virtue of the ceteris paribus clause, the premise is not committed to the implausible suggestion that the all-things-considered one should never do what will impede their well-being. (Such a thesis would rule out, contentiously, any normative requirements for sacrifice under any circumstances). Second, the clarification “*one’s*” well-being implies the premise is not making any claim about how the permissibility of our actions is restricted in the light of how they relate to *all* persons’ well-being. The idea captured here, and which I think can be simply conceded (in order to focus on more contentious aspects of the hyperagency argument) is that *all things equal,* one should refrain from doing what would probably impede one’s well-being.^12^

As a more general point—regarding P5’s place in the wider hyperagency argument—note that it is common to directly appeal at least in part to an individual’s own well-being when making evaluations of whether we ought to endorse new and emerging biotechnology—indeed, the popular welfarist approach to enhancement tells us that whether something even counts as an enhancement depends on whether it “increases [a person’s] chances of leading a good life in the relevant set of circumstances” (Savulescu, Sandberg and Kahane, 2011). There is a precedent, then, for arguing for or against certain forms of potential enhancement with explicit reference to how well-being is impacted—just as (P5) does. In sum then, I assume from here on out that if we are to reject the *Hyperagency Argument,* then, we need to look beyond (P3) and (P5).

## III. EVALUATING (P1): DO ENHANCED CAPACITIES GENERATE A CORRESPONDING INCREASE OF RESPONSIBILITIES?

Intuitively, Premise (1), which tells us that enhanced capacities make probable a corresponding increase in responsibilities, might appear just as plausible as either of (P3) or (P5). We give a child fewer responsibilities than an adult, plausibly on account of children having less developed capacities—and we generally take them to be more responsible about how their life goes as their capacities develop. Likewise, those with dementia are thought to be less than fully responsible for what they do (and thus obligated to a lesser extent) because they lack the right kind and/or degree of mental capacity for the relevant kind of reasoning.^13^ There is theoretical precedent for taking the connection between capacities and responsibility to be a tight one. The view that responsibility tracks mental capacity, *capacitarianism*, is a central underlying assumption of much of our reasoning about responsibility (Vincent, 2011, 2013).^14^

In order to appreciate the thrust of this capacitarian idea– which underlies Sandel’s P1 (on which new capacities generate new responsibilities)—it is helpful to step away from the enhancement debate specifically and consider the motivation we find for the more fundamental idea that, even when we hold all else fixed, capacity increase is plausibly a difference-maker when it comes to what responsibilities one has.

With this in mind, consider the following case, due to Vanessa Carbonell, who thinks of capacity acquisition as capable of “triggering” responsibilities one otherwise would have lacked:

...[S]uppose a man collapses on the railway platform and is dying while waiting for the paramedics. As the sole bystander I would be obligated to save his life but I do not know how. (Fortunately, the paramedics arrive just in time.) Coincidentally, a CPR course is offered at my workplace that day, and I take it. On my return commute, shockingly, another man collapses on the railway platform. No one else on the platform has the relevant knowledge, but now I do. (2013, 247)

As Carbonell sees it, in this case, an obligation has been “triggered” by the capacity you have to *do* CPR in the second version of the CPR case but not in the first. Whereas your lacking the capacity to perform CPR if you try (in the first version, by lacking the relevant know-how) insulates you from any obligation to make the attempt at CPR, your possessing it (in the second version of the case, and holding fixed everything else) seems sufficient to trigger the obligation. That is, you are plausibly *responsible* for refraining to act in the second version of the case in a way you would not be responsible for refraining to act in a scenario (like the first version of the case) where your attempts at CPR would be no more reliable than chance.

Notice now how Carbonell’s triggering point seems to hold if we run a twist on the set up of the case and shift the details so that rather than to *take the CPR* course, you instead purchase the CPR Tempo app, which offers both audio and visual cues that aid the timing of chest compressions during the process of cardiopulmonary resuscitation (CPR).^15^ As Clark et al. (2018) have suggested, if a capacity-generated obligation is generated by your gaining the capacity to do CPR via a class, it would also be generated by a capacity acquired via intelligence augmentation via the CPR Tempo app. It would at any rate, as Clark et al. suggest, seem unprincipled to diagnose the two versions asymmetrically, simply on the grounds the latter capacity is technology derived and the former is not.

Now what goes for capacity-generated obligations to others plausibly goes likewise for the kind of responsibility we have over *our* own lives.^16^ If our capacities to govern our own lives are limited, then likewise is the extent to which we are responsible for how our own lives go.

Accordingly, if the core capacitarian thesis is right, then it seems that as a person’s capacities are enhanced beyond the “normal” range, the person in possession of these capacities might in some sense become, as Vincent (2013) puts it, “hyperresponsible.”^17^ More will be rightly expected of us, both in terms of (i) the set of things we are expected to do—and expect of ourselves that we do, and (ii) the level of proficiency with which we are expected to do them.

At this point, Sandel’s P1 is looking to be on safe ground. Let us consider now a few challenges, one of which comes from experimental data. Maslen et al.’s (2015) preliminary empirical work indicates that lay people seem not to see an obvious positive link between the use of cognitive enhancement substances and an increase on responsibility or accountability. This is so *even though* there is general agreement that responsibility tracks capacity in cases like those of the child and dementia patient above. This kind of result puts some pressure on Sandel’s (P1), then—as Maslen et al. point out, “great divergence between lay and philosophical theories of responsibility puts an extra burden on the philosophers to explain why their theory is justified” (2015, 125).

One way to defend (P1) in response to Maslen et al.’s (2015) empirical results might be to embrace a kind of “error theory”—an explanation of *why* the participants surveyed tended to deny that capacitarianism extends into the enhanced range. Here, we might appeal to some of the objections to this claim—the sort of objections that those who rejected that capacitarianism extends to cover enhanced capacities might make—and then argue that these objections are not fatal. This would give us a potential diagnosis of why the view was rejected, at the same time further defending the view.

One obvious candidate for such an error theory is the main worry that Maslen et al. (2015) themselves raise about extending capacitarianism to cases of cognitive enhancement. In a nutshell, this worry is that the cognitive capacities that result from enhancement drugs and technologies are not capacities that *genuinely belong* to the enhanced person. We can see a similar sentiment reflected elsewhere in the literature, such as in the debate about whether one’s enhanced achievements are really creditable to one, a question that bioconservative ethicists such as, e.g., Leon Kass (2003) answer in the negative.

However, Maslen et al. suggest that the foregoing idea is not an intractable problem for capacitarianism—or, for our purposes, for (P1) of the hyperagency argument. At most, they argue, the objection implies that capacities that derive from “*unnatural, external* means of becoming enhanced” (Maslen et al., 2015, 126, italics mine) are not truly our own, and therefore, do not generate extra responsibilities. Here, we can assume that—by external—they mean advanced BCIs, for example, which leaves other “modest, internal” forms of cognitive enhancement—most notably, pharmacological cognitive enhancements (PCEs)—untouched. The hyperagency argument would then, at the very least, still potentially raise a serious concern about highly effective cognitive enhancement drugs.

However, regardless, there are also other defences available to the capacitarian, and thereby to Sandel in defence of (P1). The idea is that—at least as recent work in the philosophy of cognitive science (e.g., Clark and Chalmers, 1998; Palermos, 2011, 2014) suggests—*even when* capacities do in fact derive from “unnatural, external means of becoming enhanced,” it may very well still be that the capacities are *ours* in the sense that is relevant to credit attributions and reactive attitudes such as praise and blame that go handin hand with responsibility. To take one notable example often used to illustrate this idea, consider the use of a smartphone rather than on-board biomemory for the purposes of storing and retrieving information. According to proponents of extended cognition, what matters for the purposes of whether the information you have stored in the smartphone is part of *your* memory is not simply settled by asking whether the smartphone itself is something “unnatural” or “external” to your biological cognition. Rather, the relevant question is whether the smartphone is suitably *integrated* into your cognitive architecture. For Clark (2010), at least, such integration—which is not going to be satisfied in just any use of a smartphone—will require that one’s smartphone is functionally on a par with biomemory along a number of key dimensions; that is, it must be as easily accessible, the information retrieved must be default trusted, it must be easily available as needed, and the information (as with biomemory) must have been previously endorsed. In short, as Clark sees it, if you are *using* your smartphone in a way that is functionally analogous to how you use biomemory for the task of information storage and retrieval, there is no in-principle barrier to counting the phone’s storage as part of your (extended memory), such that—when you then retrieve information from external memory -- we may then attribute that memory-based belief to *you*, and credit you in a way that is analogous to how we might credit those relying on biomemory. And what goes for smartphones, will—for proponents of extended cognition—go for other kinds of enhancement via intelligence augmentation, including, e.g., tactile-visual substitution systems, BCI brain chips, etc.

Of course, the extended cognition approach in the philosophy of cognitive science, though increasingly popular, remains contested (see, e.g., Adams and Aizawa, 2008, for some notable criticisms). I mention it here merely to sketch a potentially even stronger line one might take in insisting that the kind of reasoning one might appeal to in denying P1 by denying that capacitarianism extends to enhanced capacities does not hold water. That is, one might maintain that in principle even the most radical kinds of enhancements to our capacities could generate an influx of responsibilities, so long as the external artifacts that feature in the enhancements are suitably integrated into one’s cognitive architecture by the functionalist criteria of extended cognition.

In sum, then, while there is at least some cause to doubt the capacitarian commitment underlying (P1), so too are there available defences—and, at worst, merely a concession that the target of the hyperagency argument is narrower in scope than we might first think, applying only to pharmacological cognitive enhancements.^18^ With that said, we can now turn to consider two other potential weak spots in the hyperagency argument—Premise (2) and Premise (4).

## IV. AGAINST P2: DO SUCH NEW RESPONSIBILITIES GENERATE DESIRES TO FULFIL THEM?

What about (P2), which tells us that the new responsibilities of the enhanced generate new desires to fulfill these responsibilities? For one thing, this claim is not necessarily or analytically true; it is, after all, of course *possible* that one could have additional responsibilities while at the same time—e.g., due to some kind of moral defect—simply lack the corresponding desires to fulfill them. Imagine, for example, a disillusioned CEO of a company whose shareholders have just given her more responsibilities than before. It is very possible, perhaps even realistic, that at least some of these responsibilities will not correspond with desires to fulfill them; one might be entirely *nonplussed* by the expansion of her responsibilities. Accordingly, as the worry goes, as well as not being analytically true, (P2) is also most likely not true simpliciter—there are probably individuals like the imagined CEO who have incurred absent desires to fulfill additional responsibilities.

However, these kinds of worries do not really force any major revision to Sandel’s argument, and so do not really give us substantive resources to defend cognitive enhancement against the hyperagency objection. This is for at least two important reasons. First, a weaker version of (P2)—i.e., one that makes the relationship between (i) additional responsibility and (ii) additional desires to fulfill these additional responsibilities a mere “propensity” relation weaker than an entailment relation—would actually suffice with the other premises to generate the conclusion. In a bit more detail, just consider (P3), which says “If these additional desires are not fulfilled, well-being will be impeded.” Suppose we were to tweak (P2) so that it makes explicit that the relevant relation is a propensity rather than an entailment relation; notice P3’s connection between unfulfilled desires and a depletion of well-being does not rely on the relevant unfulfilled desires being generated each time a responsibility is generated by an enhanced capacity. The claim (which we have already seen is plausible) just maintains that, ceteris paribus, unfulfilled desires impede well-being; thus, if (by a weaker version of P2) additional responsibilities (in light of enhanced capacities) generate *some* unfulfilled desires (viz., to fulfill some of those additional responsibilities), then *to this extent* (via (P3)) well-being is going to be thereby impeded.

A second point of reply to the above worry, however, takes us a bit deeper into the initial framing of the argument. *Why* would failing to live up to an explosion of new responsibilities impede well-being? There are various potential fine-grained stories here. It might be that failing to live up to one’s responsibilities diminishes one’s well-being by diminishing one’s sense of self-worth, which is displeasurable or otherwise debilitating for one.^19^ Or perhaps, the sense that one is unable to meet her new-found “hyperresponsibility” might give one a kind of “decision paralysis”^20^, where one’s own efficacy as an agent who can act on her own choices is (somewhat paradoxically) thwarted by a sense that she is in some respect not equal to the many new responsibilities she now has (for a discussion of this kind of line, see Owens, 2007). Or perhaps one is simply psychologically affected in negative ways by what Sandel describes as the additional ‘moral burden” one has to meet the explosion of responsibilities that enhancement might bring about.

What is important to note is that that the simple and relatively neutral idea that we have desires to fulfill our responsibilities that if unmet would impede well-being is broadly compatible with various of these more fine-grained explanations. Moreover, making explicit that unfulfilled desires to fulfill responsibilities impedes well-being allows us (in what is perhaps the least contentious way) to charitably construct Sandel’s hyperagency argument to get the relevant *normative* conclusion that we should (on the basis of a claimed explosion of responsibility via enhancement) not enhance our capacities; We cannot, after all, derive this conclusion from reasoning that does not include any specification of why it is that failing to fulfill responsibilities (including to oneself) negatively impacts one in a way that would be normatively strong enough to override the positive value associated with possessing new capacities via enhancement.

I mention the above to contextualize the place of (P2) in the argument in a way that allows us to better appreciate certain kinds of criticisms against it. For example, let us return to our CEO who lacks desires to fulfill the additional responsibilities, and simply imagine a more extreme version of this character—a wholesale nihilist, subsisting in a state where (except for basic bodily sensations like hunger, etc.) lacks any commitment to any values, including to the value of fulfilling any would-be responsibilities whatsoever. Should the possible or even actual existence of such a character lead the proponent of Sandel’s hyperagency argument to revise (P2)? No; this is because (as we have seen above) *so long as* there is at least some general propensity individuals have to desire to meet the responsibilities they have, making this fact in P2 explicit would offer us a suitably uncontentious way to capture a consideration about “exploded responsibilities” that would have an important *normative* bearing, and in the way that is more than sufficient for (in addition with P1, P3, and P4) generating the argument’s prescriptive conclusion. In sum, then, (P2) turns out to be much like (P1); seemingly vulnerable to objections, but such that it can be defended in light of those objections at no important cost to Sandel’s wider argumentative strategy.

That said, we return to the place of (P2) in the argument in §6. There I consider and evaluate what I take to be the most plausible way to run a variation on the Hyperagency Argument that does not rely at all on any premises about desire or desire fulfillment. That argument, we see, fares no better.

## V. AGAINST P4: ARE UNFULFILLED DESIRES REALLY SO LIKELY FOR THE HYPERAGENT?

The real weak spot is neither (P1) nor (P2) but instead (P4). Consider that if, *ex hypothesi*, our capacities were held fixed at present levels, and then additional responsibilities were incurred along with desires to fulfill these additional responsibilities, it is indeed very likely that this would result in unfulfilled desires to fulfill these responsibilities (and the ensuing lack of well-being that is implicated by such unfulfilled desires). But the situation is in fact very different when we appreciate that we are holding fixed not our present capacities but our *enhanced* capacities when considering the likelihood of desire fulfillment/unfulfillment. Even if enhanced capacities generate additional responsibilities and additional desires to fulfill these responsibilities, they also, at the same time, *make it proportionately easier* to fulfill whatever additional desires and responsibilities having one’s abilities enhanced would plausibly generate.

Compare by way of analogy: right now, you plausibly lack any *responsibility* to disarm a complicated bomb, even if it is near you and its going off would cause significant destruction^21^. However, if you were an off-duty bomb-dismantling expert and just happened to be walking by, the situation is different; such a person would plausibly have a kind of capacity-generated responsibility^22^ to dismantle the bomb that is broadly analogous to the kind of responsibility a medical doctor, but not an ordinary passenger, has on a plane to attend to an unwell passenger. These points, I take it, are in line with Carbonell’s CPR example discussed in support of the core capacitarian insight.

Now, consider this: the bomb dismantler and the doctor have additional capacity-generated responsibilities in these cases, but do these capacities at the same time generate additional unfulfilled desires they would not otherwise have had? They certainly would *if* these individuals desired to dismantle the bomb and help the passenger, respectively, but were unable to fulfill these desires. But they *are* able to fulfill them! That is, after all, precisely why we think that they have the relevant responsibilities that *they* have (but not untrained/unskilled individuals) in the first place.

The central problem with (P4), thus, is that it overlooks the sense in which there is more plausibly a kind of *symmetry* than there is an asymmetry between (i) the extent of responsibilities the acquisition of enhanced capacities would generate and (ii) the extent to which subsequent desires to fulfill these responsibilities are themselves apt to be fulfilled.

There is, however, another problem with the premise, which is that the more general reasoning of which it is an instance would seem to support, counterintuitively, the *diminishment* of existing levels of capacities. After all, if there is a positive correlation between capacities and unfulfilled desires, then it seems that, to the extent that unfulfilled desires are a detriment to well-being such that we should avoid enhancing ourselves to avoid such unfulfilled desires, we should (by parity of reasoning) militate against unfulfilled desires by actively undercutting our current capacities! However, almost no one would think there is any moral imperative to undercut our current levels of functioning. Plausibly, Sandel himself would not want to explicitly sign on to this commitment. But the challenge then becomes explaining why—in a way that is not morally arbitrary—the supposed correlation between capacities and well-being-relevant lack of desire fulfillment justifies curtailing *enhancement* of our cognitive capacities but not curtailing *present* capacities.

In sum, there are two independent reasons to reject (P4): there is a reason to do with a kind of *proportional symmetry* that can be expected between capacities generating new responsibilities and one’s capacity to meet them; and second, there is an overgeneralization worry, such that a defence of P4 would seem to imply unpalatable results about curtailing our present unenhanced abilities.

I want to close by considering and replying to potential objections to both of these reasons to reject P4. First, let us zero in on the point about symmetry. Perhaps we could envision a proponent of P4 insisting that we are overlooking something important about how enhanced abilities generate new responsibilities—in a way that might be modeled not as linear growth but as an *exponential growth,* such that each additional increase in abilities correspond with exponentially increasing levels of responsibility. On such a picture, for example, a minor enhancement might generate only some seemingly manageable new level of responsibilities, whereas a *significant* enhancement might generate responsibilities that, modeled as an exponential function, approach infinity—viz., such that we should plausibly think that with any such significant enhancement our capacity to meet these exponentially generated responsibilities would be impossible or at least highly impractical.

There is, I think, a relatively straightforward way to respond to the above point, which is to cast doubt on the idea that *even if* enhancing abilities generated *exponentially increasing* responsibilities (a claim that is itself dubious) that there is any interpretation of this idea that plausibly implies that a consequence will be *unfulfilled* responsibilities. First, a note about why the exponential growth interpretation is manifestly dubious. It is dubious because, in *unenhanced cases*, the correspondence between increases in abilities and corresponding responsibilities seems best described as linear, and in a sense that allows us to make sense of a kind of symmetrical correspondence between the two. For example, a child has less responsibility to mow the lawn than an adult. How much less? The answer seems to be: approximately as much less as the adult has a greater capacity to do it. That is, the extent to which the adult seems to have more responsibilities than the child seems to be the very same extent to which the adult has greater capacities than the child. Extrapolating from this idea: it seems that the presumption should be in favor of thinking that a similar kind of linear growth will characterize an increase in enhanced abilities and responsibilities rather than otherwise. This is the reason that the exponential interpretation is prima facie dubious. However, crucially, *even if* the relevant growth were exponential, this would seem plausible in itself only insofar as we would accept also that whatever radical enhancements generate near-infinite responsibilities also at the same time raise one increasingly toward the kind of omnipotence that would permit them to meet those abilities. Put another way, even if the proponent of P4 were granted the dubious claim that the relevant growth here is exponential, it would still be incumbent on them to explain why we should accept this claim *and* the further claim that we should *not* expect whatever high levels of enhancements would trigger near-infinite responsibilities to themselves be enhancements to abilities that give us near-infinite powers to meet them. In sum, then, the envisioned “exponential growth” style reply to the symmetry based objection to P4 does not ultimately hold up.

Even so, we might envision a proponent of P4 taking issue with the second (independent) reason given for rejecting the premise, which is that it overgeneralizes. For example, we can imagine someone might attempt to submit that there is some special feature of enhancement that makes it such that, when our abilities are enhanced, whatever corresponding responsivities that are generated by their enhancement become more difficult to fulfill. The wider idea here would be that such a point is simply inapplicable to our natural abilities, and that for this reason, the kind of overgeneralization argument against P4 (that it implies we should curtail existing abilities) does not hold up.

The worry here, though, is that attempts to resist the overgeneralization strategy in this kind of way are going to be arbitrary. At least, in the absence of a good reason to think that enhancement *as such* has an effect on abilities, whereby when abilities are increased *in that way* (rather than by traditional training, education, etc.), then the responsibilities generated by them are harder to fulfill, the default presumption should be that the strategy in P4 is going to overgeneralize. The burden of argument, is, accordingly on the proponent of P4 to explain why the reasoning here would not overgeneralize, and it is unclear to me how such an argument would not ultimately be an arbitrary one.

## VI. THE HYPERAGENCY ARGUMENT, REDUX

By this point, we have seen that even if all other premises of the argument are granted to Sandel, P4 represents a crucial hole in the argument, one that reveals a seemingly intractable problem with an attempt to reason—as Sandel does—from the capacitarian insight that responsibilities track capacities to the idea that enhancing our capacities risks as he puts it “burdening” us with too much responsibility, and in a way that makes our lives ultimately worse. The underlying problem, we have seen, is that—to the extent that we can expect enhanced capacities to generate additional responsibilities—those very same enhancements would more plausibly than not furnish us with the resources to meet whatever responsibilities the acquisition of those capacities brings about. And so the scenario on which enhanced capacities are accompanied with “too many” responsibilities is ultimately not such that it represents a risk with reference to which (all else equal) we should forbear from enhancing capacities, provided we have other good reasons to do so.

That said, I want to conclude by circling back once more to (P2). One might wonder whether there is scope to save the Hyperagency Argument by (i) noting that (P4)—the crucial premise challenged—makes reference to *desires* about fulfilling responsibilities; and (ii) then insisting that a version of the Hyperagency Argument could conceivably be run without any commitment at all to premises that reference desires.

Let us consider what such an argument might look like.

*Argument from Hyperagency (Version 2)*

(V2-P1) The enhancement of capacities makes probable a corresponding increase of responsibilities for the results of one’s choices proportionate to the degree of the enhancement.

(V2-P2) If these additional responsibilities are not met, then ceteris paribus, well-being will be impeded.

(V2-P3) It is more probable that, through the enhancement of capacities, the increased responsibilities would be unmet than met.

(V2-C1) Therefore, it is more probable that enhanced capacities, ceteris paribus, will impede well-being.

(V2-P4) If it is more probable that doing something,ϕ, will impede one’s well-being than not, then *ceteris paribus* it is not the case that one should doϕ.

(V2-C2) Therefore, *ceteris paribus,* it is not the case that one should enhance their capacities.

There are two key points to make about the above alternative version of the Hyperagency Argument. First, note that (V2-P2) requires more defence than (P2) of the original version of the argument. It leaves it incumbent on the proponent of the Hyperagency Argument to explain *why* unmet responsibilities all else equal, would impede well-being. One very weak claim here that would do the trick is the claim that we desire to meet our responsibilities, in conjunction with the observation that unfulfilled desires impede well-being. This weak claim is exactly what the original version of the argument made explicit. In this respect, the original version is meant to be a more charitable construction of the reasoning than would be this version.

However, more crucially, note that this alternative version of the argument that omits desire-based premises does not in any way eliminate the *kind* of problem that faced P4 in the original argument. Omitting desire-premises from the argument simply kicks the can down the road, in the sense that the argument relies on a premise *equally* as problematic as P4, which is premise (V2-P3), according to which it is more probable that, through the enhancement of capacities, the increased responsibilities would be unmet than met. The same kinds of reasons offered in §5 that count against P4 apply, mutatis mutandis, to V2-P3. Thus, the alternative version of the argument not only does not do better than the original version, but it seems ultimately to do worse, in that it inherits the kind of problem that faced P4 of the original argument, while also relying on a premise—(V2-P2)—which the original version characterized in a more charitable way.

## VII. CONCLUDING REMARKS

In short, I hope to have shown here, first, that Sandel draws attention to an initially arresting idea—that new powers gained via cognitive enhancement bring with them new responsibilities of a sort that is often overlooked in discussions of how enhanced capacities bear on well-being. However, as we have seen, it is not straightforward to translate this idea into a compelling argument against cognitive enhancement. In the foregoing, I have attempted to focus in on some of the subtleties that feature in a charitable construction of a Sandel-style argument that transitions from enhancement and alleged hyperresponsibility to a diminishment of well-being that would justify forgoing enhancements despite the benefits. We have seen that while there are a few premises of the argument that might initially seem contentious, the real problem with this strategy of argument lies in a mistaken idea that we should *not* be expected to meet whatever additional responsibilities we have that would be implied by enhancing our abilities. Moreover, we have seen that alternative versions of the argument seem no more promising than the original version. Of course, it might be that we *should* forego cognitive enhancements for reasons utterly divorced from considerations to do with hyperagency; equally, it might be that a very different kind of hyperagency-style argument might have more promise than one that focuses on alleged problems with responsibility explosion.^23^ However, I hope to have clarified just why it is that hyperagency arguments against enhancement that (like Sandel’s) do trade on responsibility explosion are not ultimately promising.

## Footnotes

1 See Persson and Savulescu (2008) for the origins of the debate on whether one has an “urgent imperative” to pursue moral enhancement. For some of the many responses to Persson and Savulescu, see, e.g., Fenton (2010), Harris (2011), de Melo-Martín (2018), Jotterand and Levin (2017), and Hardcastle (2018). See also Earp (2018) for a contemporary proposal that aims to overcome some of the previously highlighted issues in the moral enhancement debate.

2 This thought experiment is, of course, implausible with today’s technology. Of relevance to arguments related to hyperagency are, however, both actual and merely possible cases. Both kinds will be discussed here. For some caution though on overreliance on overstating what possibilities are currently available, see de Melo-Martín and Salles (2015).

3 See, e.g., Liao (2011) for work on enhancing the parent-child relationship and Earp et al. (2012) for arguments in favor of using “love drugs” to enhance romantic relationships when the well-being of children is at stake. More generally, see Clark et al. (2018) and Juengst and Moseley (2016) for concise summaries of some of the main considerations that bear on whether—or when—we ought to enhance ourselves.

4 For related discussion, see Earp et al. (2014).

5 For an introduction to new developments in BCI and neural implant technology, see, e.g., He et al. (2020).

6 At least, you could more easily achieve the present goals that you have. As we see shortly, Sandel maintains that the situation becomes very different once we factor in additional goals that you have on account of possessing the enhancements themselves, and the responsibilities possessing them generates.

7 For a detailed discussion on this issue, see Maslen et al. (2015).

8 An additional negative consequence of hyperagency, according to Sandel, is that it diminishes our capacity to appreciate something he takes to be valuable, which is an appreciation of ‘the gifted character of human powers and achievements’ (2012, 78). However, the negative import of this consequence would presumably be contested by an opponent of Sandel’s argument; that is, the arbitrary gifts of our original capacities are *worthy* of appreciating only if there is already something antecedently valuable about the kind of ‘chance’ Sandel notes to which we are all subject. However, the proponent of bioenhancement will find scope to potentially press back here, either by denying there is something inherently valuable about having the capacities we have by chance, or by granting this but maintaining that the value of enhancing our capacities overrides whatever value there is *inherent* in possessing the capacities we have by chance.

9 See e.g., Fletcher (2016) for a comprehensive overview of many different forms of well-being.

10 For contemporary defenses of hedonism about well-being, see, e.g., Feldman (2004), Crisp (2006), and Bramble (2016).

11 See also Dorsey (2013) and Heathwood (2005).

12 Note that the use of “impede” I am employing here is meant to be neutral with respect to substantive moral theses with respect to, e.g., consequentialism or deontology.

13 For discussion on this point, see, e.g., Peel (2013).

14 See, e.g., Fischer and Ravizza (1998), Fischer (2000) and Mele (2001) for discussion.

15 This example is due to Clark et al. (2018).

16 Granted, a strict libertarian may disagree with this presumption, though some libertarians may only reject obligations imposed by the state while accepting that there may be moral obligations one has to oneself.

17
de Sio et al. (2014) defend something similar in course of arguing that people in certain high-responsibility jobs—such as surgeons and pilots—may have a duty to use certain enhancement technologies.

18 One might wonder whether there is a limited scope to resist P1 on the basis of the possibility of potential enhancements with a very specific function—which is to cause us to better fulfill our responsibilities. One might think that the possibility of such enhancements is in tension with the idea that increasing capacities thereby increases responsibilities as per P1. However, even if there were fine-grained moral enhancements whose central function was to cause us to better meet responsibilities, this concession is compatible with the premise that *additional* responsibilities would emerge on the basis of the individual’s gaining these responsibility-fulfilling capacities. As such, the kind of case envisioned remains compatible with P1.

19 A related idea here is that hyperagency, and the corresponding level of responsibility that it engenders, might in some way negatively affect one’s sense of meaning. On this point, however, see Danaher (2014) for a useful rebuttal that aims to subvert this kind of worry by articulating some ways in which enhancement can promote rather than hinder meaning in one’s life.

20 See, e.g., Nagel (2010) for this kind of a concern; see Danaher (2014) for critical discussion.

21 For the present purposes, we can remain agnostic on the matter of whether in such a circumstance an untrained individual would have a responsibility to *attempt* to dismantle the bomb. The point here is just that it is not the case that such an individual has a responsibility to in fact dismantle it.

22 For a discussion of capacity-generated responsibilities in the special case of epistemic capacities, see, e.g., Carbonell (2013) and Clark et al. (2018).

23 A very different style of hyperagency objection, one that I have not taken up in this paper, is due to Michael Hauskeller (2011)—and which focuses moreso on the pursuit of hyperagency than on its effects. See Danaher (2014) for helpful critical discussion of Hauskeller’s proposal.
